# Individual and community-level factors associated with unmet need for contraception among reproductive-age women in Ethiopia; a multi-level analysis of 2016 Ethiopia Demographic and Health Survey

**DOI:** 10.1186/s12889-020-08653-1

**Published:** 2020-04-19

**Authors:** Melaku Yalew, Bezawit Adane, Bereket Kefale, Yitayish Damtie

**Affiliations:** Department of Public Health, College of Medicine and Health Sciences, Wollo University, Dessie, Ethiopia

**Keywords:** Unmet need, Contraceptive utilization, Multi-level analysis, EDHS 2016

## Abstract

**Background:**

There is limited evidence on the unmet need for contraceptives among married reproductive-age women especially in developing countries like Ethiopia. Thus, this study aimed to assess individual and community-level factors associated with unmet need for contraception among reproductive-age women in Ethiopia.

**Method:**

A secondary analysis was done on the 2016 Ethiopian Demographic and Health Survey (EDHS) dataset which were collected cross-sectional. A total of 9056 women who were fecund, married and/or sexually active were included in the analysis. Multi-level mixed-effect logistic regression analysis was done by STATA version 14.0 to identify individual and community-level factors. Adjusted odds ratio with 95% confidence interval was used to show the strength and direction of the association and statistical significance was declared at *P* value less than 0.05.

**Result:**

Factors significantly associated with unmet need were; ages of women between 45 and 49 years [AOR = 2.25, 95% CI: (1.34, 3.79)], greater than or equal to three living children [AOR = 1.87, 95% CI: (1.40, 2.49)], belong to richer household [AOR = 0.73, 95% CI: (0.54, 0.97)], Muslim followers [AOR = 1.37, 95% CI: (1.02, 1.83)], married more than once [AOR = 1.31, 95% CI: (1.06, 1.62)]. From community level variables: belong to the Somali region [AOR = 0.34, 95% CI: (0.19, 0.61)] were significantly associated with unmet need.

**Conclusion:**

Both individual and community-level factors were significant determinants of unmet need. From individual-level factors: advanced ages of women, many total numbers of living children, live in the richer wealth quintile, being Muslim follower and married more than once and from community-level variables: belong to the Somali region were significantly associated with unmet need for contraception. The findings suggested that health care providers should mainly focus on women nearly on menopauses, who live in the poorest household and who had many numbers of living children and married more than once to decrease the unmet need for contraceptives.

## Background

Unmet need for family planning (FP) is the number or percentage of married women who want to postpone their next birth for two or more years or stop childbearing but are not using a contraceptive method. Pregnant or amenorrheic women are also considered to have unmet need if their pregnancy was mistimed or wanted no more children [1, 2]. Married or sexually active unmarried women have unmet need for limiting if they are pregnant and didn’t want the current pregnancy at all or postpartum and didn’t want their last birth at all or fecund and want no more children. Whereas, married or sexually active unmarried women may also have unmet need for spacing if they are pregnant and wanted the pregnancy later or postpartum and wanted their last birth later or fecund and want their next child in 2 or more years or undecided on the timing. Total unmet need is the sum of unmet need for spacing and unmet need for limiting [3].

Even though FP is greatly emphasized in different strategies of maternal health, its utilization is still below what is expected and many reproductive-age women had the unmet need to it nationally as well as internationally. Globally, about 222 million couples want to stop or delay childbearing, but they are not using family planning [4]. It would range up to 84 million in Asia [5] and 200 million in developing countries [6, 7]. Again the prevalence of unmet need for contraception was 18.26% in Burkina Faso and 19.1% in Nigeria [8, 9]. According to the Ethiopian Demographic and Health Survey (EDHS) result, it was 25.3 and 22% in 2011 and 2016 respectively [10, 11].

World Health Organization (WHO) estimated that maternal death could be reduced to one third if the unmet need for FP was satisfied [12]. Globally, FP service could reduce unsafe abortion and the number of women who need medical care as a result of unsafe abortion from 5.2 million to 1.2 million and from 2.2 million to 500,000 respectively [4, 13]. Ethiopian estimates from 2005 to 2015 also showed that 24 million pregnancies were unintended. By meeting this unmet need for contraception in Ethiopia, there would be almost 6 million fewer unintended pregnancies, which lead to 2 million fewer abortions, more than 1 million under-five mortality could be averted and nearly 13, 000 maternal deaths would be decreased over ten years period [13]. A study also showed that there was an elevated risk of under-five death for children born from mothers who had an unmet need for contraception [14, 15].

Unmet need for contraception was determined by individual-level factors ((age, age of marriage education and occupation status of the mother) [16–19], husband factors (occupation and educational status of husband) [20–22], household factor (wealth)) [23] and community-level factor (place of residence, region, community-level of education and media exposure) [24–26]. Unmet need for family planning is an important concept that is largely used for reproductive health advocacy, designing family planning policies and the monitoring/evaluation of implemented programs especially Sustainable Developmental Goals (SDGs) and Growth and Transformation Plan two (GTPІІ) [27, 28].

Even though the unmet need for FP was addressed in previous studies, most of them were taking on individual-level analysis by omitting cluster effect. In the individual-level analysis, the independent assumption among clustered individuals may not work and the association at the individual level may not work at the cluster level and vice versa. So, all of these articles are subject to an atomistic or ecological fallacy [17, 21, 22, 29–39]. The factors associated with the unmet need to FP are area-specific which requires a different approach of analysis at a different level [8, 24, 40–46]. So, this study took into account those different levels of analysis and aimed to assess individual and community-level factors associated with unmet need for contraception among reproductive-age women in Ethiopia (EDHS 2016 dataset, 2019).

## Methods

### Study area and data source

**Fig. 1:**
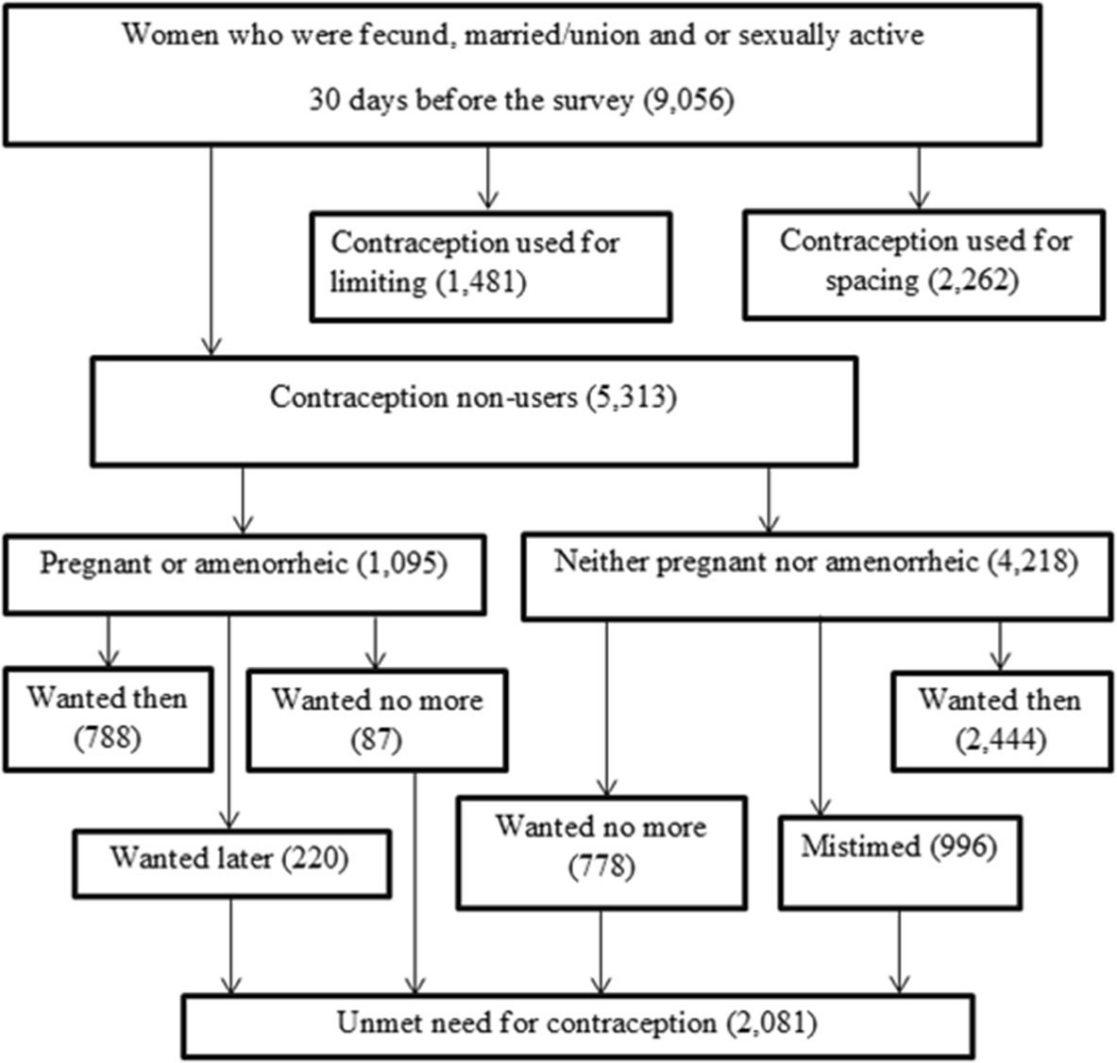
Total number of reproductive-age women who were fecund, married and/or sexually active included in the analysis in 2016 EDHS, 2019

The study was conducted in Ethiopia, which is located in the North-Eastern part of Africa, also known as the horn of Africa, lies between 3^0^ and 15^0^ North latitude and 33^0^ and 48^0^ East longitudes. This study used the EDHS 2016 dataset which was conducted by the Central Statistical Agency (CSA) in collaboration with the Federal Ministry of Health (FMoH) and the Ethiopian Public Health Institute (EPHI). Data were accessed from their URL: www.dhsprogram.com by contacting them through personal accounts after justifying the reason for requesting it. Then reviewing the account permission was given via the email. A cross-sectional study design using secondary data from 2016 EDHS was conducted. All reproductive age women who were fecund, married and or sexually active were included in the study and those women who were sexually active 30 days before the survey were excluded. A total of 9056 weighted reproductive-age women who were fecund, married and/or sexually active were included (Fig. 1).

The 2016 EDHS sample was stratified and selected in two stages. In the first stage, stratification was conducted by region and then in each region stratified as urban and rural, yielding 21 sampling strata. A total of 645 EAs (Enumeration Areas) (202 in urban areas and 443 in rural areas) were selected with probability proportional to EA size in each sampling stratum. In the second stage, a fixed number of 28 households per cluster were selected with an equal probability systematic selection from the newly created household listing.

### Variable measurement

The outcome variable for this study is dichotomized as unmet need (yes/no) which was generated from a constructed EDHS variable. It is the sum of unmet need for spacing and limiting and reproductive-age women who were married, fecund and/or sexually active have unmet needs if they don’t want any more children or want to delay their next birth for at least two years but not using contraception. Pregnant or amenorrheic women with unwanted or mistimed pregnancies or births were also considered to have unmet if they were not using contraception at the time they conceived [18, 20, 47]. Community-level variables were created by taking aggregate measures from individual-level variables in each cluster [8].

### Data processing and analysis

Data cleaning was conducted to check for consistency and missing value. Recoding, labeling, and exploratory analysis were performed by using Stata/SE version 14.0. Descriptive statistics were used to present frequencies, with percentages in tables, graphs and using texts. Sample weight was used to compensate for the unequal probability of selection between the strata that were geographically defined, as well as for non-responses.

Multilevel analysis was conducted after checking that the data was eligible for multilevel analysis that means Intra-cluster Correlation Coefficient (ICC) greater than 10% (ICC = 12.74%). Since DHS data are hierarchical, i.e. individuals (level 1) were nested within communities (level 2), a two-level mixed-effects logistic regression model was fitted to estimate both independent (fixed) effects of the explanatory variables and community-level random effects on unmet need for family planning. The log of the probability of the unmet need for family planning was modeled using a two-level multilevel model as follows:

$$ Log\left[\frac{\Pi_{ij}}{1-{\Pi}_{ij}}\right]={\beta}_0+{\beta}_1{X}_{ij}+{B}_2{Z}_{ij}+{\mu}_j+{e}_{ij} $$

Where, i and j are the level 1 (individual) and level 2 (community) units, respectively; X and Z refer to individual and community-level variables, respectively; πij is the probability of unmet need for family planning for the i^th^ women in the j^th^ community; the β’s indicates the fixed coefficients. Whereas, β0 is the intercept-the effect on the probability of the unmet need for family planning in the absence of influence of predictors; and uj showed the random effect (effect of the community on unmet need for family planning for the j^th^ community and eij showed random errors at the individual levels. By assuming each community had different intercept (β0) and fixed coefficient (β), the clustered data nature and the within and between community variations were taken into account.

During analysis first, bivariable multilevel logistic regression was fitted and variables with *p*-value less than 0.2 were selected to build the 3 models (model1–3). Then the analysis was performed in four steps: Model 0 (empty model or null model/ without explanatory variable); Model 1 (only individual-level factors) Model 2 (only community factors); and Model 3 (both individual and community-level factors). The measures of association (fixed-effects) estimate the associations between the likelihood of women to have an unmet need for family planning and various explanatory variables were expressed as Adjusted Odds Ratio (AOR) with their 95% confidence level. A variable in which its *p*-value < 0.05 was used to declare statistical significance. The measures of variation (random-effects) were reported using ICC, Median Odds Ratio (MOR) and proportional change in variance (PCV) to measure the variation between clusters.

The ICC shows the variation in unmet need for family planning for married reproductive women due to community characteristics. The higher the ICC, the more relevant was the community characteristics for understanding individual variation in unmet need for contraceptives for married reproductive women. The ICC was calculated as follows: [ICC= $\frac{\ {\delta}^2}{\ {\delta}^{2+\frac{\pi^2}{3}}}$], where *δ*^2^ is the estimated variance of clusters. MOR is defined as the median value of the odds ratio between the area at highest risk and the area at the lowest risk when randomly picking out two areas and it was calculated using the formula [MOR = exp.($\sqrt{2{x\delta}^2+0.6745}$) ≈ exp(0.95*δ*)]. In this study, MOR shows the extent to which the individual probability of having an unmet need for family planning for married reproductive women is determined by residential area. PCV measures the total variation attributed by individual-level factors and area-level factors in the multilevel model.

The presence of multicollinearity was checked among independent variables using standard error at the cutoff point of _±_2 and there was no multicollinearity. The log-likelihood test was used to estimate the goodness of fit of the adjusted final model in comparison to the preceding models (individual and community level model adjustments).

## Result

### Socio-demographic characteristics of respondents

The total numbers of women, who were fecund, married and/or sexually active and included in the analysis were 9056. The mean (SD) ages of respondents were 29.68 and 7.42 years respectively. Almost two-thirds (59.12%) of women were not formally educated. Coming to the place of residence, 7565 (83.53%) women were rural dwellers and 3759 (41.51%) women were Orthodox followers (Table 1).

### Individual and community-level factors associated with unmet need for contraception **(fixed-effects)**

After adjusting for individual and community-level factors (model 3) age of women, the total numbers of living children, a wealth of household, religion, married more than once and region were found to have a statistically significant association with unmet need for contraception.

Those women whose ages between 45 and 49 years were 2.3 times more likely to have an unmet need for contraception than those ages between 15 and 19 years [AOR = 2.25, 95% CI: (1.34, 3.79)]. The odds of unmet need for women who had greater than or equal to three living children were, almost 1.9 times higher than those who had less than three children [AOR = 1.87, 95% CI: (1.40, 2.49)]. Those women who belong to the richer household were 27% less likely to have an unmet need for contraceptive as compared to the poorest [AOR = 0.73, 95% CI: (0.54, 0.97)]. The odds of unmet need for contraceptive for women who follow Muslim were 1.4 times more likely then compare to Orthodox followers [AOR = 1.37, 95% CI: (1.02, 1.83)]. Those women who married more than once were 1.3 times more likely to have an unmet need for contraceptive than married once [AOR = 1.31, 95% CI: (1.06, 1.62)]. Lastly, those women who belong to the Somali region were 66% less likely to have an unmet need for contraceptive than Addis Ababa [AOR = 0.34, 95% CI: (0.19, 0.61)] (Table 2).

### Random effect (a measure of variation)

The results of multilevel logistic regression for random effects showed that there was a significant variation in the unmet need for contraception across the clusters (Table 3). The Intra-cluster correlation coefficients showed that 12.74% of the variation in unmet need for contraceptives was related to community-level factors. The full model also showed that there is a statistically significant variation in unmet need to contraceptive across communities or clusters. About 33.33% of unmet need to contraceptives in clusters was explained in the full model. Besides, the MOR confirmed that the unmet need for contraceptives was attributed to community-level factors. The MOR for unmet need for contraceptive was 1.79 in the empty model which indicated that there was variation between communities (clustering) (1.79 times higher than the reference (MOR = 1)). The unexplained community variation in unmet need for contraceptives decreased to MOR of 1.46 when all factors were added to the model. This showed that when all factors are considered, the effects of clustering are still statistically significant in the full models.

**Table 1:** Socio-demographic characteristics of reproductive-age women in Ethiopia using 2016 EDHS, 2019

Variable	Category	Unweight n (%)	Weighted n (%)
Age of women in years	15–19	675 (7.88)	626 (6.91)
	20–24	1779 (20.77)	1690 (18.67)
	25–29	2178 (25.43)	2343 (25.87)
	30–34	1711 (19.98)	1908 (21.07)
	35–39	1311 (15.31)	1400 (15.46)
	40–44	662 (7.73)	760 (8.39)
	45–49	248 (2.90)	329 (3.63)
Age of marriage in years	< 18	5067 (59.93)	5, 596 (62.34)
	> 18	3388 (40.07)	3380 (37.66)
Education of women	No education	4737 (55.31)	5354 (59.12)
	Primary	2471 (28.85)	2670 (29.48)
	Secondary	830 (9.69)	633 (6.99)
	Higher	526 (6.14)	399 (4.40)
Education of husband	No education	3625 (43.67)	3958 (44.81)
	Primary	2720 (32.77)	3390 (38.38)
	Secondary	1070 (12.89)	855 (9.69)
	Higher	885 (10.66)	628 (7.11)
Occupation of women	Not working	4648 (54.27)	4664 (51.50)
	Working	3916 (45.73)	4392 (48.50)
Occupation of husband	Not working	759 (9.06)	631 (7.09)
	Government employee	5341 (63.77)	6368 (71.62)
	Merchant	687 (8.20)	627 (7.06)
	Laborer	1109 (13.24)	906 (10.19)
	Others^a^	479 (5.72)	359(4.04)
Place of residence	Urban	2213 (25.84)	1491 (16.47)
	Rural	6351 (74.16)	7565 (83.53)
Region	Tigray	856 (10.08)	589 (6.54)
	Afar	730 (8.60)	81 (0.90)
	Amhara	1020 (12.02)	2189 (24.33)
	Oromia	1149 (13.54)	3488 (38.76)
	Somali	795 (9.37)	267 (2.97)
	Benishangul Gumz	675 (7.95)	95 (1.06)
	SNNP	1053 (12.40)	1877 (20.86)
	Gambela	606 (7.14)	25 (0.28)
	Harari	490 (5.77)	21 (0.23)
	Dire Dewa	506 (5.96)	44 (0.49)
	Addis Ababa	609 (7.17)	321 (3.57)
Religion	Orthodox	3190 (37.25)	3759 (41.51)
	Protestant	1537 (17.95)	1965 (21.70)
	Muslim	3673 (42.89)	3101 (34.24)
	Others^b^	164 (1.91)	231 (2.56)
Wealth	Poorest	2474 (28.14)	1734 (19.27)
	Poorer	1298 (15.29)	1832 (20.36)
	Middle	1179 (13.89)	1813 (20.15)
	Richer	1120 (13.19)	1721 (19.13)
	Richest	2418 (28.48)	1897 (21.09)

Others^a^ = who didn’t know at the time of interview and recorded as others in EDHS primarily, Others^b^ = Catholic and traditional religion followers

**Table 2:** Multi-level mixed effect logistic regression on unmet need for contraception among reproductive-age women in Ethiopia, EDHS 2016 dataset, 2019

Individual-level variables	COR (95% CI)	Model 0 ICC = 12.74%	Model 1 AOR (95% CI)	Model 2 AOR(95%CI)	Model 3 AOR (95% CI)
**Age of women**					
15–19	1		1		1
20–24	0.89 (0.63, 1.26)		0.91 (0.64, 1.30)		0.91 (0.63, 1.29)
25–29	1.15 (0.84, 1.59)		0.98 (0.67, 1.42)		0.97 (0.66, 1.42)
30–34	1.49 (1.04, 2.15)		1.09 (0.70, 1.69)		1.07 (0.68, 1.66)
35–39	1.87 (1.29, 2.73)		1.27 (0.80, 1.99)		1.25 (0.79, 1.98)
40–44	2.34 (1.58, 3.46)		1.54 (0.93, 2.54)		1.51 (0.91, 2.51)
45–49	3.53 (2.28, 5.46)		2.28 (1.37, 3.80)		2.25 (1.34, 3.79)*
**Age of marriage**					
< 18 years	1		1		1
> 18 years	0.79 (0.66, 0.95)		0.91 (0.75, 1.11)		0.90 (0.74, 1.10)
**Husband occupation**					
Not working	1		1		1
Gov’t employee	0.89 (0.65, 1.23)		0.92 (0.66, 1.29)		0.90 (0.64, 1.27)
Merchant	0.51 (0.33, 0.79)		0.67 (0.42, 1.07)		0.68 (0.43, 1.10)
Labourer	0.61 (0.42, 0.89)		0.76 (0.51, 1.13)		0.79 (0.53, 1.18)
Others*	1.05 (0.65, 1.71)		1.39 (0.83, 2.34)		1.44 (0.85, 2.44)
**Education status of a mother**					
Not educated	1		1		1
Primary	0.76 (0.64, 0.90)		1.23 (0.98, 1.55)		1.23 (0.97, 1.53)
Secondary	0.52 (0.36, 0.74)		1.11 (0.71, 1.76)		1.15 (0.72, 1.84)
College and above	0.36 (0.24, 0.55)		0.88 (0.55, 1.42)		0.94 (0.58, 1.53)
**Education status of husband**					
Not educated	1		1		1
Primary	0.72 (0.59, 0.86)		0.92 (0.76, 1.12)		0.88 (0.72, 1.08)
Secondary	0.49 (0.37, 0.65)		0.86 (0.61, 1.22)		0.84 (0.59, 1.19)
Higher and above	0.54 (0.37, 0.79)		1.09 (0.69, 1.72)		1.09 (0.69, 1.74)
**Number of living children**					
< 3	1		1		1
> 3	2.28 (1.89, 2.73)		1.88 (1.42, 2.49)		1.87 (1.40, 2.49)*
**Wealth**					
Poorest	1		1		1
Poorer	0.91 (0.73, 1.14)		0.99 (0.78, 1.26)		0.92 (0.72, 1.17)
Middle	0.78 (0.60, 1.01)		0.86 (0.65, 1.13)		0.79 (0.60, 1.04)
Richer	0.68 (0.52, 0.89)		0.78 (0.59, 1.03)		0.73 (0.54, 0.97)*
Richest	0.47 (0.36, 0.63)		0.67 (0.46, 0.99)		0.68 (0.44, 1.05)
**Visit health facility in the last 12 months**					
No	1		1		1
Yes	0.77 (0.65, 0.92)		0.87 (0.73, 1.02)		0.86 (0.72, 1.02)
**Distance to a health facility**					
Big problem	1		1		1
Not a big problem	0.77 (0.63, 0.93)		0.88 (0.72, 1.08)		0.96 (0.77, 1.18)
**Religion**					
Orthodox	1		1		1
Protestant	0.97 (0.75, 1.27)		0.92 (0.69, 1.23)		0.74 (0.53, 1.03)
Muslim	1.54 (1.24, 1.93)		1.43 (1.12, 1.82)		1.37 (1.02, 1.83)*
Others**	1.93 (1.15, 3.24)		1.94 (1.12, 3.38)		1.50 (0.84, 2.69)
**Married more than once**					
No	1		1		1
Yes	1.39 (1.13, 1.72)		1.27 (1.02, 1.56)		1.31 (1.06, 1.62)*
**Media exposure**					
No	1		1		1
Yes	0.70 (0.57, 0.86)		0.90 (0.71, 1.14)		0.89 (0.71, 1.13)
**Community-level variables**					
**Place of residence**					
Urban	1			1	1
Rural	2.24 (1.82, 2.75)			1.59 (1.06, 2.38)	1.35 (0.84, 2.16)
**Region**					
Addis Ababa	1			1	1
Tigray	1.74 (1.28, 2.36)			0.87 (0.59, 1.27)	0.88 (0.5, 1.37)
Afar	1.70 (1.22, 2.38)			0.79 (0.52, 1.22)	0.61 (0.35, 1.05)
Amhara	1.55 (1.15, 2.08)			0.72 (0.49, 1.06)	0.67 (0.42, 1.05)
Oromia	3.21 (2.40, 4.28)			1.50 (1.02, 2.20)	1.43 (0.89, 2.27)
Somali	1.17 (0.82, 1.67)			0.52 (0.33, 0.81)	0.34 (0.19, 0.61)*
Benishangul Gumze	2.37 (1.72, 3.26)			1.12 (0.75, 1.66)	1.02 (0.64, 1.62)
SNNP	2.18 (1.60, 2.95)			0.98 (0.66, 1.47)	1.19 (0.75, 1.91)
Gambela	2.50 (1.75, 3.58)			1.46 (0.94, 2.27)	1.64 (0.99, 2.70)
Harari	2.23 (1.57, 3.16)			1.37 (0.95, 1.96)	1.27 (0.81, 2.00)
Dire Dewa	2.07 (1.40, 3.07)			1.34 (0.90, 1.98)	1.12 (0.69, 1.82)
**Continuous variables**	**Coefficient** **(95% CI)**			**Coefficient** **(95% CI)**	**Coefficient** **(95% CI)**
Community level of media exposure	−0.006(−0.011, −0.002)			0.99 (0.99, 1.00)	0.99 (0.99, 1.00)
Community level of education	−0.014(−0.018, −0.010)			0.99 (0.98, 1.00)	1.00 (0.99, 1.01)

* = significant at 5%, SNNP = Southern nation, nationalities and peoples, Others* = who didn’t know at the time of interview and recorded as others in EDHS primarily, Others** = Catholic and traditional religion followers

**Table 3:** Measure of variation on individual and community level factors among reproductive-age women in Ethiopia, EDHS 2016 dataset

Measure of variation	Model 0 (Null model)	Model 1	Model 2	Model 3 (Full model)
Variance	0.48	0.42	0.33	0.32
Explained variance (PCV %)	Reference	12.50	31.25	33.33
Median odds ratio (MOR)	1.79	1.67	1.48	1.46
The intra-cluster correlation coefficient (ICC) in %	12.74	11.33	9.12	8.87
**Model fitness**				
Log-likelihood	− 4940.50	− 4633.80	− 4887.24	− 4595.49

## Discussion

As described in model 3 ages of women, the total number of living children, a wealth of household, religion, married more than once and the region was statistically significant with an unmet need for contraception. In this study, the age of women between 45 and 49 years was positively associated with the unmet need for contraceptives. The finding of this study is in line with previous studies conducted in different parts of Ethiopia [17, 47]. But, it is inconsistent with a study conducted in Enemay district, Ethiopia [36]. This discrepancy may be due to sample size and study design variation as it was conducted with a small sample size and comparative cross-sectional. It is also consistent with studies conducted in Kenya, Nigeria and Saudi Arabia [18, 48, 49]. The possible reason for this association may be due to those women near to menopause may perceive as low risk to the pregnancy.

The finding also indicated that the unmet need for contraception was negatively associated with the total number of living children. This finding is similar to previous studies conducted in Oromia and Southern nations, nationality, and Peoples Region, Ethiopia [17, 26]. It is also similar to different studies conducted in African countries [8, 18, 50, 51]. Again it is in line with studies conducted in India [20, 24]. The possible reason for this may be due to that, as the total number of living children increased their interest to have further children may be decreased. This means, those women who had many numbers of living children may have more likely to utilize FP service (more likely to have a meet need). Again it might be due to fear of child death for women who had few numbers of children [17].

Similarly, wealth also has a negative relationship with the unmet need for contraception. The finding is in line with a study conducted in Ethiopia [47]. This is also consistent with the findings from different studies conducted in Africa (Nigeria, Kenya) [23, 52, 53] Bengal, Iran, and Indonesia [16, 54, 55]. This may be due to that, wealthy women may have good health-seeking behavior and they may easily access family planning. Women who were Muslim are more likely to have an unmet need to contraceptive than orthodox followers. But, the finding of this study is contrary to the finding of a study conducted in Nigeria [49]. This difference might be due to that the sociocultural (religion) distribution and practices related to such cultures of Ethiopia and Nigeria is different.

The other findings in this study indicated those women who had undergone marriage more than once would have a high probability of unmet need to contraceptive. The finding is in line with a study conducted in Burkina Faso [51]. This might be due to that if they were married more than once, their husbands may not understand them and the women may not have the decision to control fertility. The only significant community-level variable is a region. This is also similar to previous studies conducted in Ghana and Nigeria [49, 52, 56, 57]. Despites different strengths: consider the clustering effect, using a large sample size for analysis, it is not without limitation. Since this study takes secondary data, only small numbers of community-level variables were included in the analysis as potential determinant factors for unmet need for contraception and it may also prone to recall bias.

## Conclusion

Both individual and community-level factors were significant determinants of unmet need for contraception. From individual-level factors: advanced ages of women, many total numbers of living children, live in the richer wealth quintile, being Muslim follower and married more than once and from community-level factors: belong to the Somali region were significantly associated with unmet need for contraception. The findings suggested that health care providers should extensively address women’s need through a campaign and integrate different reproductive health care services on women especially nearly on menopause, married more than once, who live in the poorest households and who had many numbers of living children to decrease the unmet need for contraceptives.

## Data Availability

The datasets used and/or analyzed during the current study are available from the corresponding author on reasonable request.

## Abbreviations

CSA: Central Statistical Agency
EA: Enumeration area
EDHS: Ethiopian Demographic and Health Survey
FP: Family Planning
ICC: Intra-cluster Correlation Coefficient
MMR: Maternal Mortality Rate
MOR: Median Odds Ratio
PCV: Proportional Change in Variance
WHO: World Health Organization

## References

[1] Westoff CF, Bankole A. The potential demographic significance of unmet need. Int Fam Plan Perspect. 1996:16–20.

[2] Takubo M: Considerations about endodontics (author's transl). Shikai tenbo= Dental outlook 1981, 58(1):125. 6945691

[3] Magure TM, Manene T, Munjanja SP, Bradley S, Mishra V (2010). Trends in unmet need and the demand for family planning in Zimbabwe.

[4] Ojakaa D (2008). Trends and determinants of unmet need for family planning in Kenya.

[5] Cleland J, Harbison S, Shah IH (2014). Unmet need for contraception: issues and challenges. Stud Fam Plan.

[6] Darroch JE, Sedgh G, Ball H (2011). Contraceptive technologies: Responding to women’s needs.

[7] Sedgh G, Hussain R (2014). Reasons for contraceptive nonuse among women having unmet need for contraception in developing countries. Stud Fam Plan.

[8] Wulifan JK, Jahn A, Hien H, Ilboudo PC, Meda N, Robyn PJ, Saidou Hamadou T, Haidara O, De Allegri M (2017). Determinants of unmet need for family planning in rural Burkina Faso: a multilevel logistic regression analysis. BMC Pregnancy Childbirth.

[9] Oginni AB, Ahonsi BA, Adebajo S (2015). Trend and determinants of unmet need for family planning services among currently married women and sexually active unmarried women aged 15-49 in Nigeria (2003—2013). Afr Popul Stud.

[10] Central Statistical Agency [Ethiopia] and ICF International (2011). Ethiopia Demographic and Health Survey 2011.

[11] Central Statistical Agency (CSA) [Ethiopia] and ICF. 2016. Ethiopia Demographic and Health Survey 2016. Addis Ababa, Ethiopia, and Rockville, Maryland, USA: CSA and ICF.

[12] WHO (World Health Organization) (2012). A guide to family planning for community health workers and their clients.

[13] Conference on Family Planning: Research and Best Practices. Munyonyo, Uganda. (2009). Retrieved from http://www.fpconference2009.orgInternational. Accessed 15-18 Nov 2009.

[14] Paudel I, Budhathoki S (2011). Unmet needs for family planning in Sunsari, eastern Nepal. Health Renaissance.

[15] Adedini SA, Odimegwu C, Imasiku EN, Ononokpono DN (2015). Unmet need for family planning: implication for under-five mortality in Nigeria. J Health Popul Nutr.

[16] Chakraborty N, Kaviraj R, Mandal A (2016). Use of family planning methods and unmet need of contraception among married women in a rural area of West Bengal: a cross-sectional study. J Dent Med Sci (IOSR-JDMS).

[17] Hailemariam A, Haddis F (2011). Factors affecting unmet need for family planning in southern nations, nationalities and peoples region, Ethiopia. Ethiop J Health Sci.

[18] Nyauchi B, Omedi G (2014). Determinants of unmet need for family planning among women in rural Kenya. Afr Popul Stud.

[19] Embafrash G, Mekonnen W (2019). Level and correlates of unmet need of contraception among women in extended postpartum in northern Ethiopia. Int J Reprod Med.

[20] Kandel N (2012). Unmet need for contraception and its associated factors among married women of reproductive age in Simichaur VDC of Gulmi District. Health Prospect.

[21] Shifa GT, Kondale M. High unmet need for family planning and factors contributing to it in southern Ethiopia: a community based cross-sectional study. Global J Med Res. 2014.

[22] Worku SA, Ahmed SM, Mulushewa TF (2019). Unmet need for family planning and its associated factor among women of reproductive age in Debre Berhan town, Amhara, Ethiopia. BMC Res Notes.

[23] Fagbamigbe AF, Afolabi RF, Idemudia ES (2018). Demand and unmet needs of contraception among sexually active in-union women in Nigeria: distribution, associated characteristics, barriers, and program implications. SAGE Open.

[24] Pal A, Yadav J, Sunita K, Singh J: Factors associated with unmet need of family planning in Bihar, India: a spatial and multilevel analysis. methods, 1:2. 10.18203/2320-1770.ijrcog20183768.

[25] Pradhan J, Dwivedi R (2015). Why unmet need for family planning remains high in Bangladesh: a community level analysis. J Womens Health Care.

[26] Bedhadha ST: Determinants of Unmet Need for Contraception among Currently Married Women in Oromia National Regional State; Evidence from Ethiopia Demographic and Health Survey Data. 2016;5(12). 10.21275/ART20163320.

[27] Assembly G: sustainable Development goals. SDGs), Transforming our world: the 2015, 2030.

[28] Health FDRoEMo: HSTP Health Sector Transformation Plan 2015/16–2019/20 (2008–2012 EFY). In.: Federal Democratic Republic of Ethiopia Ministry of Health; 2015.

[29] Tessema AL, Bishaw MA, Bunare TS (2015). Assessment of the magnitude and associated factors of unmet need for family planning among women of reproductive age group with disabilities in Bahir Dar City, Amhara region, north West Ethiopia. Open J Epidemiol.

[30] Mekonnen W, Worku A (2011). Determinants of low family planning use and high unmet need in Butajira District, south Central Ethiopia. Reprod Health.

[31] Genet E, Abeje G, Ejigu T (2015). Determinants of unmet need for family planning among currently married women in Dangila town administration, Awi zone, Amhara regional state; a cross sectional study. Reprod Health.

[32] Gebre G, Birhan N, Gebreslasie K. Prevalence and factors associated with unmet need for family planning among the currently married reproductive age women in Shire-Enda-Slassie, Northern West of Tigray, Ethiopia 2015: a community based cross-sectional study. Pan African Med J. 2016:23(1). 10.11604/pamj.2016.23.195.8386 PMC4907757 27347284

[33] Chafo K, Doyore F (2014). Unmet need for family planning and associated factors among currently married women in Misha District, southern Ethiopia: a cross sectional study. J Womens Health Care.

[34] Mota K, Reddy S, Getachew B (2015). Unmet need of long-acting and permanent family planning methods among women in the reproductive age group in shashemene town, Oromia region, Ethiopia: a cross sectional study. BMC Womens Health.

[35] Sita S (2003). Assessment of the magnitude and determinants of unmet need for family planning among currently married women in urban and Periurban Community in Awassa.

[36] Dejenu G, Ayichiluhm M, Abajobir AA. Prevalence and associated factors of unmet need for family planning among married women in Enemay District, Northwest Ethiopia: a comparative cross-sectional study. Global J Med Res. 2013.

[37] Ayalew T, Dejene A, Mekonnen Y (1995). Unmet need and the demand for family planning in Addis Ababa. Ethiop J Hlth Dev.

[38] Molla G, Belete H: Unmet need for family planning and its determinants among currently married women in Kobbo woreda, North-East of Amhara. Ethiopian Journal of Reproductive Health 2011, 5(1).

[39] Workie DL, Zike DT, Fenta HM, Mekonnen MA (2017). A binary logistic regression model with complex sampling design of unmet need for family planning among all women aged (15-49) in Ethiopia. Afr Health Sci.

[40] Berhie KA, Gebresilassie HG (2017). Multilevel logistic regression analysis of the determinants of stillbirth in Ethiopia using EDHS 2011 data. Türkiye Klinikleri Biyoistatistik.

[41] Ngome E, Odimegwu C (2014). The social context of adolescent women’s use of modern contraceptives in Zimbabwe: a multilevel analysis. Reprod Health.

[42] Khan HR, Shaw E (2011). Multilevel logistic regression analysis applied to binary contraceptive prevalence data. J Data Sci.

[43] Vu LTH, Oh J, Bui QT-T, Le AT-K (2016). Use of modern contraceptives among married women in Vietnam: a multilevel analysis using the multiple Indicator cluster survey (2011) and the Vietnam population and housing census (2009). Glob Health Action.

[44] Kaggwa EB, Diop N, Storey JD. The role of individual and community normative factors: a multilevel analysis of contraceptive use among women in union in Mali. Int Fam Plan Perspect. 2008:79–88. 10.1363/ifpp.34.079.08 18644759

[45] Ferede T (2013). Multilevel modelling of modern contraceptive use among rural and urban population of Ethiopia. Am J Math Stat.

[46] Metheny N, Stephenson R (2017). How the community shapes unmet need for modern contraception: an analysis of 44 demographic and health surveys. Stud Fam Plan.

[47] Tadele A, Abebaw D, Ali R (2019). Predictors of unmet need for family planning among all women of reproductive age in Ethiopia. Contr Reprod Med.

[48] Khalil SN, Alzahrani MM, Siddiqui AF (2018). Unmet need and demand for family planning among married women of Abha, Aseer region in Saudi Arabia. Middle East Fertil Soc J.

[49] Austin A (2015). Unmet contraceptive need among married Nigerian women: an examination of trends and drivers. Contraception.

[50] Hassan EE, Ghazawy ER, Amein NM (2017). Currently married women with an unmet need for contraception in Minia governorate, Egypt: profile and determinants. Open J Prev Med.

[51] Adebowale SA, Palamuleni ME (2014). Determinants of unmet need for modern contraception and reasons for non-use among married women in rural areas of Burkina Faso. Afr Popul Stud.

[52] Allen-Alebiosu O: A STUDY OF UNMET NEED FOR FAMILY PLANNING IN NIGERIA.

[53] Christopher A. Factors influencing regional differentials in unmet need for family planning in kenya. Nairobi. 2012;(October).

[54] Azis R, Syafar M, Zulkifli A, Seweng A: Effect of family wealth and attitudes toward unmet need for family planning among fertile couples in Makassar, South Sulawesi, Indonesia.

[55] Hosseini H, Erfani A, Bagi B. The levels and correlates of unmet need for contraception among Kurdish women in Mahabad, Iran: an application of the revised definition of unmet need.

[56] Machiyama K, Cleland J (2013). Insights into unmet need in Ghana.

[57] Oyeronke Alaba O, Olaomi JO, Olubusoye OE (2015). Spatial pattern and determinants of unmet need of family planning in Nigeria. S Afr Fam Pract.

